# The role of intraoperative neurophysiological monitoring in intramedullary spinal cord tumor surgery

**DOI:** 10.1186/s41016-023-00348-x

**Published:** 2023-11-29

**Authors:** Kai Liu, Chengyuan Ma, Dapeng Li, Haisong Li, Xuechao Dong, Bo Liu, Ying Yu, Yuxiang Fan, Hongmei Song

**Affiliations:** Department of Neurosurgery, Bethune First Hospital of Jilin University, Changchun, Jilin China

**Keywords:** Intramedullary spinal cord tumor, Intraoperative neurophysiological monitoring, D-wave

## Abstract

Intramedullary tumors are a class of central nervous system tumors with an incidence of 2 to 4%. As they are located very deep and frequently cause postoperative neurological complications, surgical resection is difficult. In recent years, many surgeons have performed electrophysiological monitoring to effectively reduce the occurrence of postoperative neurological complications. Modern electrophysiological monitoring technology has advanced considerably, leading to the development of many monitoring methods, such as SSEPs, MEPs, DCM, and EMG, to monitor intramedullary tumors. However, electrophysiological monitoring in tumor resection is still being studied. In this article, we discussed the different monitoring methods and their role in monitoring intramedullary tumors by reviewing previous studies. Intratumorally tumors need to be monitored for a summary of the condition of the patient. Only by using various monitoring methods flexibly and through clear communication between surgeons and neurophysiological experts can good decisions be made during surgery and positive surgical results be achieved.

## Introduction

Intramedullary spinal cord tumors (IMSCT) are rare and account for about 2–4% of tumors of the central nervous system [1]. Several essential fiber tracts and neural circuits are densely packed in the spinal cord. Usually tumors are located very deep, it’s frequently causing postoperative neurological complications. As the tumor grows, severe symptoms of tumor compression may occur, such as impairment of limb movement. Because there are several important fiber tracts and neural circuits distributed in the spinal cord, they are mainly divided into ascending fiber tracts and descending fiber tracts. The ascending fiber tracts are mainly composed of spinothalamic tracts responsible for transmitting pain, temperature and touch pressure sensations, spinocerebellar tracts responsible for regulating movement and posture, and thin and cuneate tract responsible for transmitting deep sensations and fine skin touch. The descending fiber tracts mainly include the corticospinal tract, rubrospinal tract, vestibulospinal tract, reticulospinal tract, tectospinal tract, and medial longitudinal fasciculus. Among them, the corticospinal tract that controls the movement of the trunk and limbs is more susceptible to surgery. Due to the narrow space in the spinal cord and the compression of the tumor, various neurological symptoms caused by compression or damage of fiber bundles are more likely to occur during tumor removal, such as limited limb movement and sensory loss. In severe cases, spinal shock may even occur, threatening the patient’s life. The surgical removal of IMSCT might damage the fiber tracts leading to severe consequences. The goal of neurosurgery is to maximize tumor resection and minimize the incidence of neurological complications. For this, several intraoperative neurophysiologic monitoring (IONM) techniques have been developed for neurosurgical procedures. IONM consists of multimodal technologies that allow for intraoperative neurological integrity assessment through techniques such as somatosensory-evoked potentials, motor-evoked potentials, D-waves, and neuroelectromyography.

Initially, orthopedic surgeons implemented evoked potential monitoring of the spinal cord to reduce neurological morbidity [2]. Only somatosensory-evoked potentials (SSEPs) were available at that time. However, because SSEPs could only monitor sensory pathways, the damage to the motor pathways remained undetected when SSEPs were used alone. Thus, the extent of the postoperative motor deficit could not be predicted [3]. Furthermore, SSEPs can be defined as signal averaging, and there is a delay between when the signal changes and when those changes are recorded.

Since SSEPs cannot monitor the motor pathways, motor-evoked potential (MEPs) monitoring is used to predict the motor function. There are two ways to evaluate the integrity of the motor pathways, i.e., by using epidural electrodes (D-wave) or using signals from limb muscles (muscle MEPs), or a combination of both. The main monitoring modalities include the use of somatosensory-evoked potentials (SSEPs), transcranial motor-evoked potentials (TCMEPs) via limb muscles or spinal epidural space (D-waves), and dorsal column mapping (DCM). Thus, many of these monitoring modalities can monitor the integrity of the fiber tracts and neural circuits to predict the prognosis of the patients.

In this article, we reviewed studies in the field of IONM and discussed its role in IMSCT surgery.

### SSEPs

Somatosensory-evoked potentials (SSEPs) can be used to monitor the dorsal column-medial lemniscus pathway that performs tactile discrimination, vibration sensation, form recognition, and joint/muscle sensation (conscious proprioception) [4]. The dorsal columns are vascularized by the paired posterior spinal arteries [5]. Usually, the cortical and subcortical SSEPs are elicited by stimulation of the median nerve at the wrist and the posterior tibial nerve at the ankle (intensity: 40 mA, duration: 0.2 ms, and repetition rate: 4.3 Hz). The SSEPs are recorded using corkscrew-like electrodes inserted in the scalp (CS electrode, Nicolet Biomedical, Madison, WI, USA) at CZ′-FZ′ (legs) and C3′/C4′-FZ (arms), according to the 10–20 international electroencephalogram system. Baseline measurements are recorded three times. The baseline measurement signals are recorded when the patient undergoes general anesthesia and at the beginning of the application of monitoring equipment, when the patient is in the supine position; subsequently, the patient is placed in the prone or lateral position, depending on the procedure, and just before skin incision [6]. The baseline acquisition time also applies to the following operations.

The usual warning criteria for SSEPs are a 50% reduction in signal amplitude or 10% prolongation of latency from baseline [7]. Experiments conducted using SSEPs might differ in sensitivity and specificity. In a study by Hyun, the sensitivity and specificity were found to be 75% and 50%, respectively [8]. Because most surgeries of IMSCT are performed dorsally, the damage to the sensory pathway is more probable, which can cause changes in the SSEPs signals. But in a study by Skinner, the sensitivity and specificity were 80% and 100%, respectively [9]. Thus, SSEPs can help to monitor the function and integrity of neural circuits. Tumors in different locations may have different effects on SSEPs. Some studies have shown that the specificity of thoracic spine tumors is higher than that of cervical spine tumors [10]. However, SSEPs have some limitations, for example, they require averaging, which prolongs the acquisition of results and delays the initiation of surgery. This might increase the risk of neurological damage. Furthermore, because SSEPs monitor only sensory pathways, injury to the motor pathways is not detected, which can lead to detecting “false-negative SSEPs”; especially when SSEPs are used alone, false-negative results are more probable. Such “false-negative SSEPs” are often used incorrectly to determine the occurrence of a postoperative motor deficit, although intraoperative SSEPs remain unchanged. A true false-negative scenario occurs when postoperative sensory deficits occur but are not predicted by intraoperative SSEPs changes. The loss of SSEPs during the initial posterior longitudinal myelotomy is often momentary, and the amplitude of SSEPs can recover before the end of the procedure when, after tumor removal, the dorsal columns are no longer laterally displaced [11].

Some researchers have used spinal cord evoked potentials after electrical stimulation to the brain instead of MEPs to monitor intraoperative motor function and have achieved promising results [12, 13]. Under such circumstances, changes in the intraoperative sensory function cannot be monitored probably because the sensory pathway might be damaged while slit the spinal cord surface. SSEPs can monitor the changes in the sensory function of patients by monitoring the integrity of the column-medial lemniscus pathway. The changes in the SSEPs waveform can also help to determine the best position to cut the spinal cord. Opening and pulling might cause waveform changes, signal loss, and the inability to continue monitoring. However, no new methods have been developed to monitor sensory functions. Thus, SSEPs are still preferred for monitoring the sensory function of the patients.

The alarm principle of SSEPs and its sensitivity specificity in past studies.

**Table:** 

Study	Warning criteria	Intramedullary spinal cord tumor patients’ number	Sensitivity	Specificity
Park et al. [14]	A 50% decline in amplitude	26	47	82
	An all-or-none criterion	26	24	97
Park et al. [15]	A more than 10% of N20 or P30 latency prolongation	31	54	71
Hyun et al. [8]	An amplitude reduction of 50% of baseline values and latency increases 10%	17	75	50
Ille et al. [16]^a^	A 50% decline in amplitude or latency increases > 10%	71	69	71
Jin et al. [17]^a^	A 50% decline in amplitude	25	100	57
	An all-or-none criterion	25	50	86
Siller et al. [18]	An amplitude ≥ 50% and/or an increase in SSEPs latency ≥ 2 ms	24	60	75
Lakomkin et al. [19]	A persistent (over 3 or more recordings) loss of ≥ 50% in the amplitude	17	78	88

^a^For combined application of MEPs, SSEPs, fEMG

### DCM

DCM can be used to identify anatomic landmarks to avoid the “dorsal column dysfunction” syndrome. The midline of the normal spinal cord is the sulcus between the elevated posterior columns and midway between the root entry zones. However, due to spinal cord edema, capillary neovascularization, arachnoid scarring, and rotation of the cord (caused by intramedullary tumors), the midline of the normal spinal cord might be altered significantly. In such cases, DCM can be used to solve the issue [20]. The DCM method can help the surgeon find midline between dorsal columns based on SEP amplitude gradient measurements recorded directly from the surgically exposed dorsal columns. DCM was performed with a microscale multi-contact electrode consisting of 8 parallel wires, each 76 I in diameter, spaced 1 mm apart, and embedded in silicon. This electrode is capable of recording subtle differences in SEP amplitude between recording surfaces following tibial nerve stimulation. The location of the maximum SEP amplitude recorded after stimulation of the left and right tibial nerves tends to be where the midline is. Mehta et al. found that DCM can decrease the rate of postoperative posterior column dysfunction [21]. By using DCM, the new postoperative posterior column dysfunction decreased significantly to 9%, compared to the 50% in standard therapy [21]. By using DCM, surgeons can localize the physiological midline and determine a safe location to cut the spinal cord, thus reducing the postoperative rate of posterior column dysfunction.

### MEPs

#### Muscle MEPs: multipulse technique

Muscle MEPs (mMEPs) are useful for monitoring motor pathways [22]. These spinal cord motor tracts are located in the anterior spinal cord. The anterior spinal artery supplies blood to the ventral columns, which are responsible for sensations of pain and temperature [5]. Muscle MEPs are elicited by transcranial electrical stimulation using a multipulse technique, where short trains of five square-wave stimuli (single pulse duration: 0.5 ms, interstimulus interval: 4 ms, rate: 2 Hz) are provided through corkscrew electrodes placed at the C1/C2 (lower limbs) and C3/C4 (upper limbs) scalp sites [23]. Based on the law of motor function downlink, neuroelectrophysiologists investigate the MEPs monitoring mode to monitor the integrity of the motor downlink pathway. The traditional MEPs occur due to a three-level exchange of motor fibers and other reasons. Many issues arise while measuring MEPs, such as unstable waveform and effects of anesthesia, and thus, it is not as specific as D-wave. However, it still plays a “cornerstone” role in monitoring contemporary intramedullary tumors.

The main criteria used for monitoring include all or none, amplitude, threshold, and morphology. The all-or-none criterion is considered to be very important. Its theoretical basis is pathophysiology, such as compression or traction that can disturb many corticospinal axons in the compact spinal cord leading to ischemia, which can rapidly disable the lower motor neurons. Due to the conduction of multiple synapses, mMEPs are nonlinear; thus, disproportionately large decrements can result from small reductions in the number of conducting corticospinal axons or lower motor neuron excitability. Furthermore, other descending, ascending, or propriospinal systems that support the excitation of lower motor neurons might disrupt the process of tumor removal. This might also reduce the firing of lower motor neurons and mMEPs despite an intact corticospinal pathway [24]. Thus, the all-or-none criterion is sufficient to monitor the condition of the spinal cord during IMSCT [25]. Regarding the amplitude for spinal cord monitoring, at least an 80% decrease in the amplitude can result in a positive prediction [26]. The threshold is defined as a significant increase in the stimulating currents for a specific duration to maintain MEPs signals [27]. While monitoring the condition of the spinal cord, a threshold elevation of ≥ 100 V is considered to be the warning criterion [28, 29]. Morphological changes in MEPs waveforms are often used in conjunction with threshold elevation as a warning criterion. Such changes include the transformation from polyphasic long-duration potentials to biphasic short-duration potentials and are combined with ≥ 100-V threshold elevation [30].

The alarm principle of MEP and its sensitivity specificity in past studies.

**Table:** 

Study	Warning criteria	Intramedullary spinal cord tumor patients	Sensitivity	Specificity
Park et.al [14]	A 50% decline in amplitude	26	47%	82%
	The all-or-none criterion	26	24%	97%
Hyun et al. [8]	A 50% decline in amplitude	31	100%	25%
Ille et al. [16]	A 50% decline in amplitude	71	76	69
Jin et al. [17]	A 50% decline in amplitude	25	100%	57%
	The all-or-none criterion	25	50	86
Kothbauer et al. [31]	The all-or-none criterion	100	100	91
SillerI et al. [18]	An 80% decline in amplitude	24	50	60
Kurokawa et al. [32]	A 20% decline in amplitude	58	70	77
Kim et al. [33]	A 20% decline in amplitude	22	Not mentioned	Not mentioned
Muramoto et al. [34]	A 70% decline in amplitude	80	96	49
Lakomkin et al. [19]	A 60% decline in amplitude	17	38	100

Generally, a true positive (TP) refers to an MEPs alert with a persistent decrease in the number of potentials at the end of the operation, followed by a new neurological motor deficit after the operation. A false positive (FP) refers to an MEPs alert with a persistent decrease in the number of potentials at the end of the operation and the absence of any new postoperative deficit. A true negative (TN) refers to the absence of MEPs alerts during surgery and no new postoperative deficits. A false negative (FN) refers to the absence of an MEPs alert with a new postoperative motor deficit. Current recommendations for warning criteria while monitoring intraoperative evoked potential are empirically derived [35]. When the alarm threshold is adjusted to 50% or 70–80%, it increases the false-positive and false-negative rates. Thus, adjusting the alarm threshold can also affect the intraoperative FP and FN. False negatives might arise when MEPs cannot detect segmental spinal cord injury when the muscles from which the MEPs are recorded are different from the innervated muscle arising from the spinal anterior horn cell exposed to the risk of injury due to the localization of the spinal cord tumor and the operation. Additionally, there is no standard for prognostic evaluation. What degree of functional decline might be considered to be a positive signal is not known. For example, there is no standard to determine mild postoperative paralysis as a positive sign or moderate or more severe dyskinesia. Similarly, there is no standard regarding the time to assess the prognosis of patients. For example, patients who experience motor dysfunction after surgery and recover after 3 months may be classified into different categories according to different standards [32].

**Table:** 

Possible causes of false negatives
I. The design of the alarm threshold will affect the false-negative rate
II. The muscles recorded by MEPs are different from the innervated muscles produced by the actually damaged spinal cord anterior horn cells
III. There is no uniform standard for prognostic evaluation
IV. There is no uniform standard of the time to assess the prognosis of patients

False positives might arise because intact fast-conducting corticospinal tract fibers can immediately compensate for an injury to the corticospinal tract. Thus, no clinical motor deficits are present postoperatively [36]. The completion of a movement requires the simultaneous balance of the sensory and motor functions. It not only involves the corticospinal tract but might also need the co-participation of the tectospinal, rubrospinal, reticulospinal, and vestibulospinal tracts [37]. However, MEPs only monitor the function of the corticospinal tract, which might be one of the reasons for false-positive outcomes. Improper selection of monitoring methods can also lead to false-positive and false-negative outcomes. Additionally, FPs might arise when the anesthetic fades, especially when using a high propofol dose, low blood pressure, low body temperature, or the compression of inguinal artery in the prone position.

**Table:** 

Possible causes of false positive
I. The design of the alarm threshold will affect the false-negative rate
II. The damaged corticospinal tract is compensated by the fast-reacting fiber, and it is finally shown as a false
III. MEPs only monitor the function of the corticospinal tract, and cannot monitor the other pathways involved in the formation of movement
IV. The selection of monitoring method is inappropriate
V. Other reasons during the operation, such as high dose of propofol, hypotension, hypothermia, or compression of the inguinal artery in the prone position

MMEPs have several aspects, for example, they can be used to monitor signal changes in different groups of muscles. Unlike other monitoring models, mMEPs can be used to monitor lower sacral roots, including the sphincter. Furthermore, mMEPs have high sensitivity and a low false negative [38]. They do not require averaging, and thus, when the amplitude exceeds the threshold, surgeons can take appropriate measures quickly. However, mMEPs cannot be applied to all patients. Occasionally, scarring from prior surgery can hinder the insertion of electrodes [39]. Furthermore, mMEPs have some limitations. For example, it can be hindered by a serious single nerve root injury, which decreases the efficacy after the administration of inhalational anesthetics and paralytic agents.

#### Direct-wave: single-pulse technique

The direct (D)-wave is a direct measure of the number of functioning fast-conducting fibers in the corticospinal tract [23]. The D-wave is elicited by a single-pulse stimulating technique (0.5 ms duration) and is recorded from the epidural or subdural spaces of the spinal cord [40]. Eicker et al. placed a ventral subdural electrode during anterior cervical spine surgery [41]. D-waves above the 50% cutoff value could predict the long-term functional deficiency of motor control in the lower extremities. The D-wave has several advantages. For example, it is not influenced by blood pressure, heart rate, temperature, and anesthetics. Sala et al. found that when a change in the D-wave amplitude is below 50%, patients always exhibit transient paraplegia, even if the mMEPs are completely lost. However, if the D-wave disappears during an operation, patients always exhibit permanent paraplegia postoperatively [42]. Thus, D-wave is a highly specific predictor of postoperative motor deficits. Because of its anatomical properties, nerve fibers are found to decrease craniocaudally and are absent in the lumbosacral region. Thus, D-wave cannot be applied to monitor the spinal cord below T10–T11. D-waves need midline recording, and thus, they cannot differentiate which side of the limb is damaged. The D-wave is considered to be the gold standard for assessing the integrity of the corticospinal tract in spinal monitoring [43]; however, no study has mentioned this. Although D-wave also has some limitations, it can record more accurate, stable, and real-time data than MEPs by monitoring fast-response fibers. It can improve the degree of tumor resection and predict and prevent the loss of new motor functions. Thus, it can improve the prognosis of patients. Additionally, it has a high specificity and can prevent the transformation of “surgery-related transient paraplegia” to irreversible paralysis. D-waves are also more sensitive to severe spinal cord injuries.

**Fig. 1:**
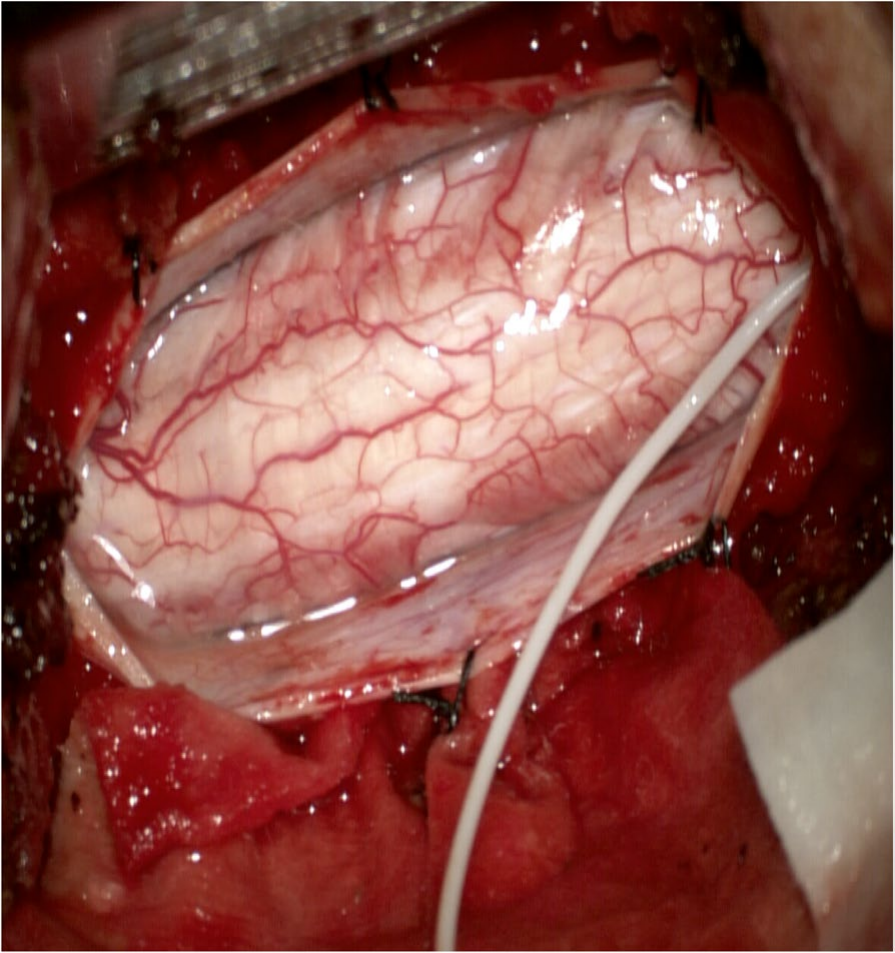
Use of D-wave in intramedullary tumor surgery

**Fig. 2:**
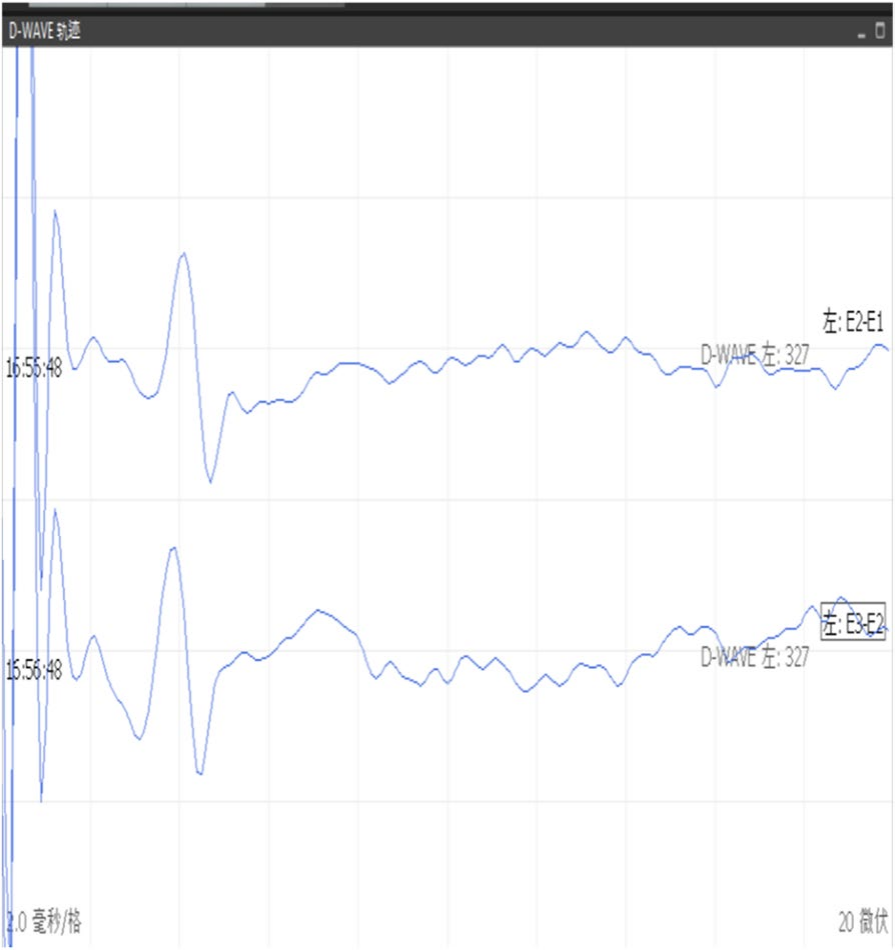
Intraoperative monitoring images

The amplitude change of D-waves is correlated with the postoperative outcome. If the D-wave remains unchanged, even though the mMEPs are lost, there is no permanent deficit in the postoperative function. If the D-wave amplitude falls below 50% of the baseline value or even disappears, patients often have permanent paraplegia [44–46]. However, motor functions might be impaired even if the D-waves remain unchanged. For example, when hemiplegia occurs in patients on one side before surgery due to the lesions of one cerebral hemisphere, intraoperative D-waves may be normal. The D-waves might originate from the healthy hemisphere [47]. Because the D-wave is produced from white matter tracts, motor deficits occurring due to gray matter injury might not alter D-waves [48]. Furthermore, as the gray matter is more sensitive to ischemia than the white matter, D-waves are less sensitive to ischemia than mMEPs [49]. Additionally, radiotherapy may limit the performance of D-waves. In patients who were administered radiation therapy, D-waves were not detected at the beginning of surgery even when mMEPs could be recorded. This might be because radiation therapy damages conductivity in the long tracts of the spinal cord [50]. Alternatively, it could either be due to the desynchronization of the descending activity through the corticospinal tract, reflected by the D-wave, or because electrodes cannot be placed due to dural adhesions caused by radiation therapy (Figs. 1 and 2).

### Electromyography

#### Spontaneous electromyography

Spontaneous or free-running electromyography (frEMG) is a technique where needle electrodes are placed in the muscles of interest to monitor the corresponding nerve roots [4]. By recording the frEMG activity, which includes spikes, bursts, or trains, it can monitor elaborate signal changes in muscle groups to predict the condition of the corresponding nerve roots. Free-running EMG is crucial for detecting early abnormalities before detection by mMEP. It has a high negative predictive value (98%) but is limited by low specificity and sensitivity with muscle-relaxing anesthetics [51]. Free-running EMG can be used with SSEPs and TcMEPs but cannot be used alone. Skinner et al. showed that abnormalities detected by free-running EMG could predict postoperative motor deficits with a sensitivity of 87.5% and specificity of 83.3%. However, frEMG is sensitive to temperature changes, treatment with cold water, cauterization, and the use of a high-speed drill. In such cases, false frEMG will be activating [52]. Since the main application of frEMG generally is the monitoring of nerve root function, in most cases, its role in intramedullary tumors that do not involve nerve roots might not be ideal.

### Bulbocavernosus reflex

The bulbocavernosus reflex (BCR), a reflex evoked by genital stimulation, is recorded from the anal sphincter muscle [53]. A BCR is elicited by the electrical stimulation of the genitals (dorsal penile/clitoral nerve). In male patients, a pair of surface electrodes are placed on the proximal (cathode) and distal (anode) parts of the penis. In female patients, the electrodes are placed on the clitoris (cathode) and the labia (anode). The afferent pathways of BCR include the sensory fibers of the pudendal nerves, while the efferent pathways include the motor fibers of the pudendal nerves and the anal sphincter muscles. The S2–S4 is the center of this reflex. Therefore, BCR can be used to monitor the integrity of urinary function during spinal tumor surgery [54]. During spinal cord surgery, the most important influencing factor of the BCR might be the operation [55, 56]. Specifically, the severity of the preoperative symptoms related to the urinary system has the greatest impact on the changes in the waveform of BCR during surgery [57]. There are currently no standard warning criteria for BCR. Nobuito et al. suggested that a 75% reduction in the BCR amplitude might be considered as the warning criterion. This preliminary clinical report on the warning criteria for the BCR might improve the safety of the patient during surgery [55]. The warning criteria of BCR and its relationship with sphincter control, sphincter-detrusor dysfunction, and sexual function need further investigation [58]. BCR monitoring is primarily conducted to protect the integrity of the urinary function of the patients. Using BCR can greatly reduce the chance of damaging the urinary function of a patient undergoing high-level intramedullary tumor resection. Therefore, in intramedullary tumor resection, BCR has not been widely used. However, the application of BCR might become indispensable during the resection of low-lying tumors, such as T12 intramedullary tumors or conus medullary tumors.

### Triggered EMG

In triggered EMG or compound muscle action potentials (CMAPs), the intact cortical bone should be electrically insulated for a well-placed pedicle screw from the adjacent nerve root. The technique can also be used to determine the degree of loosening of filum terminale in lipoma of filum terminale [59]. By using a monopolar, the intensity is set at 1–2 mA for fibrous/lipomatous tissue and reduced to 0.2–0.5 mA for nerve roots to maintain consistency in morphology, low variability of the CAMP waveform, and identical muscle group activation across multiple trials at the same stimulation site for identifying and separating the nerve and the tumor [59]. Because the fatty filum is devoid of any neural tissue, by using trEMG, mapping may help to identify a filum terminale in nerve roots [60]. It can also help to identify hidden sacral nerve roots intermingled with lipomas of filum terminale [61]. However, because of “functional filum terminale” or the presence of neural elements in the filum, the role of trEMG in lipomas of filum terminale needs further investigation [62]. Similar to frEMG, performance in intramedullary tumor resection might be limited due to the mechanism of action of trEMG.

### Multimodal IONM

Each monitoring method has its own advantages and disadvantages. In actual work, multiple monitoring methods are often used together. When combined with other monitoring approaches, the accuracy of IONM increases [12]. If each monitoring method is used alone, various neurological complications may occur due to false positives or false negatives, causing serious consequences. SSEPs is commonly combined with MEPs or other modalities. Then, DCM or EMG is used according to the specific situation. Multimodal IONM allows the neuromonitoring team to detect the injury in the spinal tracts early. Combining SSEPs and MEPs increases the sensitivity and decreases the specificity of monitoring. With increased sensitivity, signal changes can be detected early, which can facilitate rapid measurements and reduce the risk of postoperative neurologic deficits substantially. However, this might lead to very early interruption of surgery and decrease the degree of tumor resection, resulting in premature termination of the surgical procedure. At this time, the combined use of multimodal detection can effectively improve the specificity of detection, thereby avoiding the problem of low tumor resection caused by premature termination of surgery due to a single signal change. To achieve maximum tumor resection while preserving the patient’s neurological function.

In most cases, mMEPs disappear first, then the D-wave amplitude either stabilizes or drops. In such cases, the patient always shows transient postoperative paraplegia, and most patients recover completely within a few hours to weeks [63]. However, if the D-wave decreases by more than 50% along with a decrease in mMEPs, and if early appropriate measures are not taken during surgery, the patient always shows permanent paraplegia postoperatively [31]. This is called “surgically induced transient paraplegia,” which might be caused by the reversible inactivation of the noncorticospinal descending tracts and the propriospinal system, while the fast-conducting corticospinal fibers remain intact. Very rarely does the D-wave amplitude decrease without significant changes in the mMEPS. This is because the wound occurs due to vascular injury rather than direct damage to the physical tissue [39]. The exact reason needs to be determined.

### Combining neurosurgery with IONM

Not only does neuromonitoring need to be supervised by a team of neurophysiologists, but it also needs the coordination of the team conducting surgery. Once the monitoring amplitude is changed, IONM neurophysiology considers whether the change in the amplitude needs to be processed first, if neurophysiologists decide that the changes in the amplitude need to be dealt with according to the warning criteria. IONM should be performed by practitioners skilled at both technical and interpretative aspects of monitoring so that quick responses to changes can be made and conveyed in real time to the operating team. Three factors have been identified to promote signal recovery, including blood pressure, irrigation, and time [49].

The first factor is time. When the monitor amplitude changes, the resection is paused until the amplitude recovers. Generally, the waiting time is 30 min; longer waiting times can increase the risk of surgery and the cost. Second, warm saline solution irrigation during surgery can solve the problem of accumulation of potassium in the extracellular space that can block conduction, thereby preventing blockage of nerve impulse transmission and clearing out irritating products and metabolites in the blood [64, 65]. Additionally, increasing the mean arterial pressure or using papaverine can also improve perfusion to counteract incipient ischemia. MEPs are correlated with blood pressure, and sustained hypotension might affect MEPs [66]. The mean arterial blood pressure can increase if intraoperative monitoring signals decrease [67]. When necessary, the surgeon must decide between keeping the residual tumor by terminating the procedure earlier and the problems that might arise in the patient by continuing the procedure. Alternatively, the surgeon might conduct staged surgery. However, when a change in the signal occurs during intramedullary tumor resection, the surgical intervention to rescue the signal has a low success rate. In IMSCT, the spinal cord is aggravated by the tumor. By removing the tumor from the spinal cord, the small blood vessels that enter the spinal cord and tumors are blocked from the segmental artery and the anastomotic branch to reduce the blood flow [68]. The efficacy of TcMEPs for IMSCT is debatable [69].

Not all methods can be used for all cases of IMSCT surgery. Appropriate monitoring methods should be selected for different situations.

Besides its application in surgery, IONM is used in the diagnosis and treatment of intramedullary tumors.

 IMSCT are rare tumors and account for about 2–4% of tumors of the central nervous system. The surgery for intramedullary tumors is challenging and often difficult. With the development of electrophysiological technology, the role of IONM in surgeries has become very important. Neuromonitoring is an important predictor of postoperative function and can “prevent” irreversible neural damage by facilitating proper intervention after the IONM alert. It also helps to determine how much of a tumor to remove. By using multimodal IONM, fiber tracts and neural circuits can be provided effective protection. Ghadirpour et al. found that after applying IONM (SSEPs, mMEPs, and D-wave) in 68 patients, 7.35% of the patients showed dramatic changes; malignant postoperative neurological deficit was avoided by timely processing [70]. Korn et al. found that the application of multimodal IONM in patients (*n* = 100) demonstrated a high level of accuracy; the sensitivity and specificity were 82% and 95%, respectively [71]. These studies showed that IONM can effectively improve the prognosis of patients. IONM can also influence the extent of resection. Skinner et al. found that the application of IONM in patients with no significant changes in intraoperative deficits resulted in a total resection without new postoperative dysfunctions [9].

IONM might not be required for all surgeries involving spinal cord tumors. Previous studies have shown that total resection of ependymoma can result in a good prognosis [72]. For ependymoma, since the boundary between the tumor and the spinal cord is distinct, it is easier to perform complete tumor resection for ependymoma than for other intramedullary tumors, such as astrocytoma. Therefore, it is less likely to damage the spinal cord when tumors that have a clearly defined surgical plane are removed. Additionally, for intramedullary hemangioblastoma, if the hemangioblastoma is not removed completely, the residual tumor might rebleed or swell, thus occupying the intramedullary space and causing adverse effects. Hence, even if there are neurological deficits after surgery, the tumor must be completely removed. Also, for some tumors that only require biopsy followed by radiotherapy and chemotherapy, such as high-grade spinal cord tumors, IONM is not required. IONM has certain risks, and the requirements for anesthesia, operation time, cost, etc. are relatively high. Therefore, in specific circumstances, a good prognosis is possible by removing the tumor without electrophysiological monitoring [73–75]. Some studies have shown that monitoring or not testing is not significantly helpful during total tumor resection [42]. Therefore, whether IONM needs to be performed during the resection of all tumors is debatable, and further research is required.

## Data Availability

The datasets used and/or analyzed during the current study are available from the corresponding author on reasonable request.

## Abbreviations

IMSCT: Intramedullary spinal cord tumors
IONM: Intraoperative neurophysiologic monitoring
MEPs: Motor-evoked potentials
TcMEPs: Transcranial motor-evoked potentials
DCM: Dorsal column mapping
SSEPs: Somatosensory-evoked potentials
mMEPs: Muscle motor-evoked potentials
TP: True positive
FP: False positive
TN: True negative
FN: False negative
frEMG: Spontaneous or free-running electromyography
BCR: Bulbocavernosus reflex
CMAPs: Compound muscle action potentials
EMG: Electromyography
